# Sex differences in pulmonary arterial hypertension: role of infection and autoimmunity in the pathogenesis of disease

**DOI:** 10.1186/s13293-018-0176-8

**Published:** 2018-04-18

**Authors:** Kyle A. Batton, Christopher O. Austin, Katelyn A. Bruno, Charles D. Burger, Brian P. Shapiro, DeLisa Fairweather

**Affiliations:** 1 Department of Cardiovascular Medicine, Mayo Clinic, Jacksonville, FL USA; 2 Department of Pulmonary and Critical Care Medicine, Mayo Clinic, Jacksonville, FL USA

**Keywords:** Pulmonary arterial hypertension, Sex differences, Autoimmune disease, Inflammation, Systemic sclerosis, Myocarditis

## Abstract

Registry data worldwide indicate an overall female predominance for pulmonary arterial hypertension (PAH) of 2–4 over men. Genetic predisposition accounts for only 1–5% of PAH cases, while autoimmune diseases and infections are closely linked to PAH. Idiopathic PAH may include patients with undiagnosed autoimmune diseases based on the relatively high presence of autoantibodies in this group. The two largest PAH registries to date report a sex ratio for autoimmune connective tissue disease-associated PAH of 9:1 female to male, highlighting the need for future studies to analyze subgroup data according to sex. Autoimmune diseases that have been associated with PAH include female-dominant systemic sclerosis, systemic lupus erythematosus, rheumatoid arthritis, Sjögren’s syndrome, and thyroiditis as well as male-dominant autoimmune diseases like myocarditis which has been linked to HIV-associated PAH. The sex-specific association of PAH to certain infections and autoimmune diseases suggests that sex hormones and inflammation may play an important role in driving the pathogenesis of disease. However, there is a paucity of data on sex differences in inflammation in PAH, and more research is needed to better understand the pathogenesis underlying PAH in men and women. This review uses data on sex differences in PAH and PAH-associated autoimmune diseases from registries to provide insight into the pathogenesis of disease.

## Background

Pulmonary arterial hypertension (PAH) is characterized by an elevated resting mean pulmonary arterial pressure of ≥ 25 mmHg in the presence of a normal pulmonary capillary wedge pressure of ≤ 15 mmHg [1]. Vasoconstriction of the small pulmonary arteries increases pulmonary vascular resistance leading to right heart failure and premature death [2]. Narrowing and occlusion of the vessel lumen is due largely to endothelial cell proliferation and vascular remodeling [3]. Central to the pathology of PAH is the plexiform lesion which is comprised of a proliferation of endothelial cells and vascular smooth muscle cells (VSMC), myofibroblasts, and inflammatory cells that occlude the pulmonary arteries. Pulmonary hypertension is currently divided into five diagnostic groups based on etiology. PAH is classified as group 1 and includes idiopathic, heritable (previously termed familial), drug- or toxin-induced, and PAH associated with other conditions, as well as persistent pulmonary hypertension of the newborn [4]. Conditions associated with PAH include autoimmune diseases (particularly connective tissue diseases), congenital heart disease, portopulmonary hypertension, HIV infection, and *Schistosoma mansoni* infection leading to schistosomiasis. Factors that may lead to vasoconstriction in PAH include genetic predisposition, sex hormones, infections, autoimmune diseases, inflammation, and/or immune complex deposition. This review compiles data on sex differences in PAH and PAH-associated autoimmune diseases from registries and how this information may provide insight into the pathogenesis of disease.

## Sex differences in the epidemiology of PAH

Most cardiovascular diseases (CVDs) like atherosclerosis, myocardial infarction, myocarditis, and dilated cardiomyopathy occur predominantly in men [5–7]. One exception is PAH [8–10]. Table 1 lists the sex ratio for PAH reported from a number of registries [11–20]. These data indicate a female predominance in PAH of around 2–4 over men for all races and ethnicities and across all ages that have been studied to date except for HIV-associated and portopulmonary hypertension, which tend to occur more often in men [17, 21, 22].

**Table 1:** Female to male ratio in major PAH registries

Time period	Registry	Number	Median age (years)	Overall F:M ratio	References
1981–1985	USA/NIH	187	36	1.7:1	[11]
1982–2006	USA	578	48	3.3:1	[12]
1986–2001	Scotland	374	51	2.3:1	[13]
1999–2004	China	72	36	2.4:1	[14]
2000–2010	Czech Republic	191	52	1.9:1	[15]
2001–2009	UK/Ireland	482	50	2.3:1	[16]
2002–2003	France	674	50	1.9:1	[17]
2006–2007	USA/REVEAL	2525	50	3.9:1	[18]
2007–2008	Spain	866	45	2.5:1	[19]
2007–2013	Europe/COMPERA	1283	68	1.8:1	[20]

Understanding sex differences in PAH subgroups may provide important information about the pathogenesis of disease. For example, the sex ratios for connective tissue disease (CTD)-associated PAH skew more strongly toward females when examined as an individual subgroup (i.e., 9:1 female to male) (Table 2). Most registries combine PAH subgroups making it difficult to ascertain percentages and sex ratios by subgroup. For this reason, future studies should report sex difference data for each subtype within PAH (Table 3). With the exception of hereditary PAH, subgroups may then additionally be combined based on sex differences (Table 3). Because most autoimmune diseases can take up to 10 years for patients to obtain a diagnosis [5, 23, 24], it is possible that some CTD-associated PAH cases are categorized as idiopathic PAH in younger patients. The pronounced gender difference that exists within PAH subgroups in epidemiologic studies indicates that more research is needed to better understand sex differences in the pathogenesis of disease.

**Table 2:** Female to male ratio in PAH subgroups

PAH type	Scotland [13]	USA [12]	France [17]	Spain [19]	USA/REVEAL [18]
*n*	374	578	674	866	2525
Overall	2.3:1	3.3:1	1.9:1	2.5:1	3.6:1
Idiopathic	1.7:1	3:1 (with familial)	1.6:1	2.7:1	4.1:1
Heritable		3:1 (with idiopathic)	2.3:1		
Congenital	2.1:1	2.1:1	2:1	2.7:1	
Anorexigen-associated		All female	14.9:1		
CTD-associated	4.8:1	6.7:1	3.9:1	8.8:1	9:1
HIV-associated		1:7.7	1:1.2	1.5:1	
Portopulmonary hypertension		2.3:1	1:1.5	1:1	
Toxic oil syndrome				2.3:1	
Veno-occlusive disease				4:1

**Table 3:** Analysis of PAH according to sex, separate from genetic PAH

Genetic PAH	ᅟ
Heritable PAH	
Congenital PAH	
Female-dominant PAH	
Heritable PAH: estrogen-driven BMPR2 polymorphisms	
Idiopathic PAH	
CTD-associated PAH	
Rheumatic autoimmune disease-associated PAH (i.e., SSc, systemic lupus erythematosus, rheumatoid arthritis, myositis/ dermatomyositis, Raynaud’s syndrome, CREST, Sjögren’s syndrome)	
SSc-associated PAH/lcSSc (female-dominant form of SSc)	
Other female dominant autoimmune diseases associated with PAH (i.e., thyroiditis)	
Anorexigen-associated PAH	
Male-dominant PAH	
HIV-associated PAH with myocarditis (myocarditis is male dominant and can be caused by HIV or *Schistosoma mansoni* infection)	
SSc-associated PAH/dcSSc (male-dominant form of SSc)	
Portopulmonary hypertension^a^	

*Abbreviations*: *BMPR2* bone morphogenic protein receptor 2; *CREST c*alcinosis, *R*aynaud’s syndrome, *e*sophageal dysmotility/gastroesophageal reflux disease, *s*clerodactyly, and *t*elengiectasias; *dcSSc* diffuse cutaneous SSc; *lcSSc* limited cutaneous SSc; *PAH* pulmonary arterial hypertension; *SSc* systemic sclerosis

^a^Not all studies support male predominance [17, 18, 22]

## Female-dominant autoimmune diseases associated with PAH

The reported gender difference for CTD-associated PAH ranges from 4:1 to 9:1 female to male (Table 2). The two largest registries that reported sex differences for PAH subgroups, REVEAL and the Spanish Registry of Pulmonary Arterial Hypertension, found a sex ratio for CTD-associated PAH of 9:1 female to male [18, 19, 21]. The Spanish Registry of Pulmonary Arterial Hypertension found that 61% of CTD-associated PAH patients had the autoimmune connective tissue disease systemic sclerosis (SSc), also referred to as scleroderma [19]. Other female-dominant autoimmune diseases or syndromes aside from SSc that have been associated with PAH include systemic lupus erythematosus, mixed connective tissue disease, myositis (also called dermatomyositis), rheumatoid arthritis, Raynaud’s syndrome, CREST syndrome, autoimmune hepatitis, Sjögren’s syndrome, and thyroiditis [8, 9, 25–31]. The sex ratio and prevalence of autoimmune diseases that may be associated with PAH are listed in Table 4 with a ratio of women to men ranging from 2:1 to 19:1 [24].

**Table 4:** Sex ratio of autoimmune diseases that may be associated with PAH [24]

Autoimmune disease	Ratio of women to men	Prevalence (per 10^5^)
Hashimoto’s thyroiditis	19:1	792
Sjögren’s syndrome	16:1	14
Systemic sclerosis/scleroderma^a^	12:1	24
Primary biliary cirrhosis	8:1	15
Autoimmune hepatitis type 2	8:1	3
Graves’ disease (thyroiditis)	7:1	629
Systemic lupus erythematosus^a^	7:1	32
Autoimmune hepatitis type 1	4:1	17
Mixed connective tissue disease	4:1	3
Rheumatoid arthritis^a^	3:1	860
Myositis/dermatomyositis^a^	2:1	5
Myocarditis^b^	1:2	–

^a^Rheumatic autoimmune diseases include Sjögren’s syndrome, systemic sclerosis/scleroderma, systemic lupus erythematosus, rheumatoid arthritis, and myositis/ dermatomyositis

^b^The prevalence of myocarditis is not known

SSc has been estimated at a female dominance as high as 12:1 (Table 4), but sex ratios range from 4.6:1 to 12:1 female to male [24, 32–34], a range that is similar to those found in CTD-associated PAH. SSc is typically diagnosed prior to menopause in women, while men with SSc are diagnosed at an older age than women [34]. The sex ratio of women to men with SSc in their childbearing years has been estimated at 15:1 while it lowers to 2.4:1 after age 50 [35, 36], indicating the importance of analyzing data by gender and age (i.e., the cutoff for menopause is around age 50–55). SSc has been divided into two clinical forms: limited cutaneous SSc (lcSSc) which occurs more often in women and diffuse cutaneous SSc (dcSSc) which occurs more often in men (Table 3) [34, 37, 38]. Men with SSc (i.e., dcSSc) were found to have more severe disease, progress rapidly, and have a higher incidence of PAH with an increased risk of heart failure and death [37]. Testosterone is able to increase inflammation and remodeling in the myocardium (i.e., myocarditis and dilated cardiomyopathy) and vessel wall (i.e., atherosclerosis) leading to an increased rate of heart failure and death in men [6, 7, 39]. Women with the SSc (i.e., lcSSc) have more autoantibodies and CREST syndrome, develop less severe disease, and have a lower mortality [34]. The sex difference in disease presentation of SSc reflects the sex differences observed in men and women in general, with more women developing autoantibodies/autoimmune diseases and reduced mortality, while more men develop heart disease (i.e., atherosclerosis, myocarditis) and are at an increased risk of heart failure and death [6, 7, 40] (Table 5).

**Table 5:** Summary of key concepts

Women	Men
Increased PAH (2–4:1 women to men)	PAH lower in men
PAH peaks pre-menopause when estrogen highest	Increased HIV-associated PAH in men with myocarditis, which is also higher in men
Estrogen increases DCs, T cells, Th2 response, Treg, TGFβ:Th2, and TGFβ promote fibrosis	Testosterone increases mast cell and macrophage inflammation
Estrogen increases B cells, autoantibodies, and ICs	Testosterone increases TLR4 signaling, which promotes inflammation and fibrosis
Estrogen decreases BMPR2 expression on immune cells resulting in increased TGFβ and lung fibrosis	Testosterone increases profibrotic IL-1β
Increased CTD-associated PAH (9:1 women to men), especially SSc-associated PAH	More men have dcSSc form of SSc (organ involvement)
Estrogen increases SSc/lcSSc (skin involvement): pre-menopause SSc 15:1 women to men	SSc in men associated with decreased survival
SSc in women associated with increased survival	SSc in men associated with increased lung fibrosis
Estrogen decreases lung fibrosis in women with SSc	
Estrogen increases Raynaud’s syndrome	

Elevated right heart pressures were found in 59% of patients with SSc, CREST syndrome, and systemic lupus erythematosus using exercise stress echocardiography to evaluate the change in pulmonary arterial pressure [41]. Importantly, it has been estimated that over 50% of mortality in patients with SSc is due to PAH and interstitial lung disease, with PAH alone accounting for around 30% of deaths [42, 43]. Although SSc-associated PAH was found to have a female to male ratio of 5.5:1, men had more rapid PAH progression, interstitial lung disease, dcSSc (the male-dominant clinical form of SSc), and a decreased 1-, 2-, 3-, and 5-year survival compared to women [38]. This is similar to what is known for PAH cases in general where the incidence of PAH is higher in women but 5-year survival is worse in men [19, 20, 44]. Interestingly, cardiovascular disease occurs in nearly all SSc patients [45]. In addition to PAH, SSc patients have a higher risk of developing arrhythmias, coronary artery disease (CAD), myocarditis, pericarditis, cardiomyopathy (including dilated cardiomyopathy), and heart failure [5, 37, 45–51]. SSc has been reported to be an independent risk factor for CAD with accelerated atherosclerosis in these patients [49]. Overall, cardiac involvement in SSc is more common in dcSSc patients, where both dcSSc and CVD occur more often in men [50]. Similarly, CAD, myocarditis, pericarditis, cardiomyopathy, and heart failure all occur more often in men [5–7].

SSc is a systemic autoimmune disease characterized by inflammation, remodeling, fibrosis, immune complex (IC) deposition, thrombosis, and vasculopathy that can either primarily affect the skin (i.e., scleroderma) or internal organs (i.e., SSc) like the lungs, gastrointestinal tract, and kidneys [29, 40]. SSc is part of a group of female-dominant autoimmune diseases that include myositis/dermatomyositis, rheumatoid arthritis, systemic lupus erythematosus, Raynaud’s syndrome, CREST syndrome, and Sjögren’s syndrome. All of these autoimmune diseases have been associated with PAH (reviewed in [5, 8, 9, 24–26]) [52]. Women with one autoimmune disease often have evidence of other autoimmune diseases with overlap occurring especially between rheumatic autoimmune diseases which include SSc, systemic lupus erythematosus, rheumatoid arthritis, Sjögren’s syndrome, and myositis/dermatomyositis (Table 4). Up to one fifth of SSc cases have features of rheumatoid arthritis, systemic lupus erythematosus, and/or myositis [53]. Interestingly, women with Raynaud’s syndrome, rheumatoid arthritis, and thyroiditis often also have Sjögren’s [54–56]. Sjögren’s syndrome in particular causes inflammation, remodeling, and fibrosis of the lung [51, 57].

**Fig. 1:**
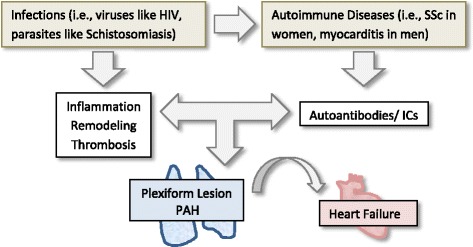
Hypothesis for how infections and autoimmune diseases may promote PAH in women and men. Infections and autoimmune diseases like HIV and systemic sclerosis (SSc) are able to cause inflammation, immune complex (IC) deposition, and remodeling in the lung that may lead to pulmonary arterial hypertension. Estrogen increases the risk of developing autoimmune diseases like SSc following infection or other insults by promoting antibody/autoantibody and IC deposition that may contribute to the increased incidence of PAH in women, especially for autoimmune diseases that affect the lung-like SSc. PAH associated with HIV infection occurs more often in men and HIV-associated PAH patients also have myocarditis. Inflammatory mechanisms that drive myocarditis in men following infection may also drive PAH

Raynaud’s syndrome frequently occurs with autoimmune diseases like mixed connective tissue disease (including SSc) (86%), systemic lupus erythematosus (31%), rheumatoid arthritis (22%), and Sjögren’s syndrome (13%) [53, 58]. The earliest clinical manifestation of microvascular dysfunction in SSc is the presence of Raynaud’s syndrome, seen in nearly 100% of SSc patients [59, 60]. Raynaud’s syndrome is associated with dysregulation of autonomic and small sensitive nerve fibers resulting in vasoconstriction of peripheral small vessels [58, 61, 62]. In patients with Raynaud’s syndrome, the presence of abnormal nail fold capillaries predicts the evolution to established SSc [63]. Anti-nuclear autoantibodies (ANA) are believed to be involved in the pathogenesis of Raynaud’s syndrome leading to IC deposition on vessels that trigger vasoconstriction [58]. Recently, ICs have been reported in the lungs of patients with PAH that were associated with perivascular macrophages and circulating monocytes [64]. Thus, autoantibodies/ICs present during autoimmune diseases like SSc, Sjögren’s syndrome, CREST syndrome, and Raynaud’s syndrome could contribute to vasoconstriction of small vessels in the lung, thereby contributing to the pathogenesis of PAH (Fig. 1).

ICs are composed of antibodies (against foreign antigen-like viruses or parasites) and/or autoantibodies (antibodies against damaged self) complexed with antigens/autoantigens and complement components (i.e., complement C3). ICs precipitate out of the blood binding to tissues when they become very large and/or sometimes when temperature changes occur, as occurs in Raynaud’s syndrome in response to cold. ICs strongly activate proinflammatory innate immune responses. Viral infections frequently lead to IC formation and may contribute to the association of viral infections with the onset/diagnosis of PAH [65]. Rheumatic autoimmune diseases like SSc, systemic lupus erythematosus, Sjögren’s syndrome, and rheumatoid arthritis are renowned for IC formation, where ICs arise from the presence of multiple autoantibodies including rheumatoid factor resulting in tissue deposition and activation of an immune response [5, 51]. Rheumatoid factor is an autoantibody formed against the Fc region of antibodies (e.g., IgM, IgG). As part of the normal immune response to infection, rheumatoid factor promotes complement fixation and clearance of ICs by macrophages, enhances antigen presentation, and amplifies the avidity of IgG, all of which protect against infections [66–68]. But the presence of rheumatoid factor predicts a more aggressive, destructive disease course for rheumatic autoimmune diseases where it is present in 70–90% of rheumatoid arthritis, 60–80% of Sjögren’s syndrome, 30% of systemic lupus erythematosus, 20% of myositis, and 20% of SSc patients [66, 67, 69]. Interestingly, HIV and *Schistosoma mansoni* infections (as well as other viral, bacterial, and parasitic infections associated with rheumatic autoimmune diseases and myocarditis) are known to form ICs with rheumatoid factor [5, 70], suggesting that CTD-, HIV-, and schistosomiasis-associated PAH may have a similar immunopathology (Fig. 1).

Autoantibodies against fibroblast, smooth muscle, and endothelial cells have been found in a significant proportion of CTD-associated PAH patients [71–73]. Of 380 PAH patients that underwent testing for the presence of autoantibodies, 33% of idiopathic and familial PAH (combined) tested positive for ANA in contrast to 94% of CTD-associated patients [12]. Out of 115 patients retrospectively identified with either idiopathic PAH (56%) or CTD-associated PAH (44%), 76% were women and ANA were detected more often in CTD-associated PAH cases [74]. The fact that ANA were detected in a large number of idiopathic PAH patients in both of these studies and that autoimmune diseases often take many years to diagnose suggest that some idiopathic PAH cases may develop CTD-associated PAH later in life [5, 23, 75].

## Male-dominant autoimmune diseases associated with PAH

Two PAH registries reported that more men than women had HIV-associated PAH [12, 17]. In a study of HIV-associated PAH patients, all had evidence of myocarditis; 12 of 15 had myocardial inflammation confirmed by biopsy (the gold standard for diagnosis), and the other three patients had myocardial fibrosis in biopsies suggestive of healed/chronic myocarditis [76]. Ten of the patients with HIV-associated PAH and myocarditis were men and five women, a sex ratio of 2:1 male to female, which is the same as myocarditis patients in general (Table 4) [6]. HIV infection causes myocarditis, which is an autoimmune disease characterized by myocardial inflammation, remodeling, and progression to dilated cardiomyopathy [5, 77]. Myocarditis is one of the few autoimmune diseases that occurs more often in men [6]. A study of 28 asymptomatic patients with chronic HIV infection found that 82% of the patients had evidence of myocarditis and myocardial fibrosis by cardiac magnetic resonance imaging [78]. However, the study did not examine whether the HIV patients had PAH.

Myocarditis is associated with SSc and other rheumatic autoimmune diseases that are associated with PAH. Of 181 newly diagnosed cases of SSc, 7 had evidence of myocarditis [48]. Compared with SSc patients, patients with SSc and myocarditis had early disease, myositis, autoantibodies against ANA, detectable virus, pericardial effusion, and systolic and/or diastolic dysfunction [48]. Ten out of 19 female SSc patients (53%) had evidence of myocarditis and cardiac remodeling [79]. Similar to PAH, myocarditis has been reported to occur with all rheumatic autoimmune diseases (i.e., systemic lupus erythematosus, rheumatoid arthritis, SSc, myositis) except for Sjögren’s syndrome [5, 80–85]. Infections that have been associated with SSc and other rheumatic autoimmune diseases like Epstein-Barr virus, parvovirus B19, cytomegalovirus, coxsackievirus, *Salmonella*, and HIV are all known causes of myocarditis [5, 76, 86, 87]. Importantly, schistosomiasis, which is associated with PAH, has also been reported to cause the eosinophilic form of myocarditis in people and animal models [88–91]. The association of myocarditis with SSc-, HIV-, and schistosomiasis-associated PAH suggests the possibility that infection may increase the likelihood of PAH in men either directly or indirectly by inducing myocardial/vascular inflammation, fibrosis, and/or IC deposition. IC deposition on the exterior of the heart leads to pericarditis and occurs in animal models of myocarditis [92] and in myocarditis patients where it is termed perimyocarditis [93].

The fact that many autoimmune diseases associated with PAH like systemic lupus erythematosus, rheumatoid arthritis, and thyroiditis do not primarily cause disease in the lungs or pulmonary vasculature suggests that immune mechanisms that drive the pathogenesis of autoimmune diseases may also be important in PAH. Cytokines that are elevated in HIV infections and myocarditis include interleukin (IL)-1β, IL-6, IL-8, tumor necrosis factor (TNF), and platelet-derived growth factor (PDGF). These cytokines drive the pathogenesis of HIV/AIDs and myocarditis, as well as PAH [6, 94, 95]. Future studies should examine whether subjects with HIV- and/or *Schistosoma mansoni*-associated PAH also have myocarditis.

## Immunopathology of PAH

The primary pathophysiology of PAH involves vasoconstriction, inflammation, and vascular remodeling [2]. The histopathologic hallmark of PAH is the plexiform lesion, which consists of endothelial cell (EC) proliferation and VSMC hypertrophy in small precapillary pulmonary arterioles surrounded by inflammatory cells and fibrosis [96]. The narrowing of the lumen by proliferation of vascular cells combined with constriction of the arteriole due to remodeling and fibrosis results in elevated pulmonary arterial blood pressure. A mixed infiltrate consisting of mast cells, macrophages, dendritic cells, and T and B cells surround plexiform lesions based on histology from PAH lung biopsies [65, 96]. The presence of antibody, autoantibody, and/or IC deposition in pulmonary vessels may further occlude vessels promoting PAH (Fig. 1).

### Endothelial cell proliferation

EC proliferation is a major component of the pathology of plexiform lesions. ECs require vascular endothelial growth factor (VEGF) for survival, and excess amounts of this growth factor induce EC proliferation [97]. PAH patients have elevated circulating levels of VEGF compared to controls and higher expression of the VEGF receptor in plexiform lesions [98, 99]. Evidence of EC activation includes elevated levels of sera vascular cell adhesion molecule-1 (VCAM-1) in PAH patients, which is elevated on ECs after proinflammatory cytokine stimulation where it recruits inflammatory cells [99]. Evidence of EC damage (and autoimmunity) includes increased sera levels of anti-EC autoantibodies in patients with PAH compared to controls [71]. However, the initiator of EC injury is not known and could include exposure to toxins (i.e., drugs/anorexigen and chemicals), infections, and autoantibodies, all of which promote IC formation. IC deposition on ECs may directly lead to vasoconstriction or indirectly promote PAH by damaging cells and recruiting inflammation.

### Vascular smooth muscle cell hypertrophy

Another key component of the pathology of the plexiform lesion includes proliferation of VSMCs. PDGF is a potent VSCM mitogen that drives hypertrophy and proliferation of this cell population during PAH [97]. PDGF levels are significantly elevated in lung tissue from PAH patients compared to control lungs [100]. Epidermal growth factor and its receptor are important for VSMC proliferation and survival and may play a role in PAH [101, 102]. Transforming growth factor (TGF)-β1 promotes collagen deposition while Toll-like receptor (TLR)4-induced IL-1β and IL-18 enhance remodeling associated with VSMCs [103].

### Fibroblast/myofibroblast activation

An important contributor to arterial constriction and elevated blood pressure in PAH patients involves vascular remodeling/fibrosis that stiffens the pulmonary arteries [2]. Pulmonary arterial adventitial fibroblasts are the major source of collagen and remodeling enzymes termed matrix metalloproteinases (MMPs) and tissue inhibitors of MMPs (TIMPs) [97, 104]. Other key growth factors and cytokines that convert fibroblasts to myofibroblasts include PDGF, fibroblast growth factor (FGF), insulin-like growth factor (IGF)-1, angiotensin II, TNF, IL-4, IL-1β, IL-17, and TGF-β1 [96, 97, 105, 106]. PDGF antagonists reverse vascular remodeling in animal models of PAH [100]. TGF-β1 stimulates fibroblast proliferation and collagen synthesis and inhibits MMP-induced degradation of collagen [106]. Angiotensin II, a component of the cardiac renin-angiotensin-aldosterone system, indirectly increases TGF-β1 in response to mechanical stretch or damage [107]. TNF, IL-1β, and IL-4 also induce fibroblast proliferation resulting in collagen synthesis and remodeling [108, 109]. IL-1β and TNF have long-lasting affects by upregulating and maintaining TGF-β1 transcription for as long as 6–10 weeks [110]. In contrast, IFN-γ directly inhibits transcription of TGF-β1 and IL-4 via signal transducer and activator of transcription (STAT)1 [111]. IFN-γ prevents fibrosis by inhibiting fibroblast proliferation, collagen synthesis, and mast cell degranulation [109, 112, 113].

### Inflammation

Lung biopsies from PAH patients reveal a mixed infiltrate consisting of mast cells, macrophages, dendritic cells, and T and B cells surrounding plexiform lesions [65, 96]. Mast cells and macrophages are a major source of proinflammatory and profibrotic cytokines that recruit other immune cells and promote vascular remodeling. Mast cells induce remodeling by releasing TNF, IL-1β, IL-4, IL-18, and TGF-β1 as well as collagen/matrix digesting enzymes like tryptase, chymase, and serpin A3n that stimulate perivascular fibroblast proliferation and collagen deposition [114–118]. Mast cell-derived tryptase induces vascular angiogenesis promoting PAH [114]. Although mast cells are typically present in small numbers surrounding vessels, they are significantly increased in patients and animal models of PAH [119, 120]. Prevention of mast cell degranulation attenuated PAH in an animal model [121], suggesting that mast cells play a role in the disease process. Mast cells act as antigen presenting cells and provide the Th2-associated cytokines IL-4 and IL-33 to drive macrophages and T cells toward an alternatively activated M2 macrophage/Th2 phenotype increasing B cells and antibody/autoantibody production [109, 117, 122].

Macrophages and dendritic cells are innate immune cells that activate the immune response and promote remodeling (reviewed in [122]). Remodeling and a B cell-induced antibody/autoantibody response require a T helper-type 2 (Th2) immune response that is initiated by IL-4 and/or IL-33 during antigen presentation. *Schistosoma mansoni* parasitic infections are strong inducers of M2 macrophages and Th2 responses that are associated with elevated IL-4, IL-13, and TGF-β1 [123]. In contrast, HIV infection requires an IFN-γ/Th1-type immune response for viral clearance but is known to skew the immune response to a M2/Th2 response as a strategy to evade the immune system [124]. M2 macrophages are also associated with autoimmune diseases because they scavenge damaged self-tissues following injury through the mannose receptor and scavenger receptor-A where they increase autoimmune disease by presenting self-tissue to T cells [122]. IC-mediated activation of M2 macrophages and mast cells results in the release of profibrotic mediators like IL-1β, TGF-β1, fibronectin, and MMPs.

### Immunogenetics

Genetic defects/polymorphisms in the bone morphogenic protein receptor (BMPR)2/TGF-β signaling axis have been identified with a predisposition for PAH [125]. Today, over 300 different polymorphisms in BMPR2 are known to exist and are present in approximately 75% of individuals with a family history of PAH [125]. However, only 10–20% of carriers of polymorphisms in BMPR2 develop PAH [126, 127], indicating that environmental factors are critically important for disease development. Polymorphisms in BMPR2 and other members of the TGF-β superfamily such as ALK1 and SMAD9 have been associated with elevated growth factors and proinflammatory mediators that contribute to remodeling during PAH [73, 128, 129]. BMP and TGF-β have also been found to synergistically activate Foxp3+ regulatory T cells (Tregs) [128]. Tregs reduce inflammation and are critical in regulating or preventing autoimmune diseases [130]. Thus, dysfunctional BMPR2 signaling may lead to reduced Treg which could increase inflammation and the risk of developing autoimmune diseases associated with PAH [131]. Aggregations of lymphocytes, resembling lymphoid follicles, have been detected in the lung tissue of PAH patients suggesting local chronic immune stimulation [128, 132]. Thus, genetic predisposition contributes to the development of PAH and may be more closely linked to autoimmune-associated PAH than previously realized.

## Sex hormone effects on inflammation and remodeling

Microarray, proteomics, and other molecular tools have revealed how sex hormones and chromosomes regulate the immune response under normal and pathologic conditions [7, 118, 133, 134]. Estrogen receptor (ER)-α, ER-β, the androgen receptor, and other steroid hormone receptors are expressed on or within endothelial cells, VSMCs, and fibroblasts in mice and humans [6, 129, 130]. Similarly, human and mouse dendritic cells, monocytes, macrophages, mast cells, and T and B cells can express ERα, ERβ, and/or the androgen receptor (reviewed in [129, 131–138]). It is important to emphasize that ERα, ERβ, and the androgen receptor are present on/in tissue and immune cells in both men and women where they function essentially as growth factors maintaining the normal physiology of cells. Cells in women should have a higher ratio of ERs to androgen receptors on/in cells and visa versa for men. In response to foreign- or self-antigens, sex hormone receptors on tissue and immune cells alter the immune response in a sex-specific manner. The effect of sex hormone receptors on individual immune cells also depends on the context of the response. For example, the dose of the hormone and whether hormone receptor signaling occurs predominantly on the surface of the cell or within the cytoplasm alters the downstream effect of sex hormones [134, 139]. In general, females have a more robust T and B cell response and correspondingly lower burden of bacterial, viral, and parasitic infections [137].

ERα has been found to promote dendritic cell maturation and antigen presentation to T cells [140–144]. In contrast to estrogen, testosterone has a largely negative impact on dendritic cells, although the effect is complex and depends on the hormone concentration [134]. ERα, ERβ, and the androgen receptor are expressed on the membrane surface and within mast cells and macrophages [39, 40]. Testosterone increases mast cell and macrophage numbers in the heart of males with myocarditis and upregulates cardiac TLR4, IL-1β, and IL-18 [118, 145, 146]. Estrogen inhibits TNF, IL-1β, and IL-6 from macrophages by downregulating NFκB [147–151]. Estrogen has also been found to skew macrophages to an alternatively activated M2 phenotype by its ability to increase Th2-type cytokines like IL-4 [5, 134, 152]. Estrogen signaling through ERβ in monocytes upregulates expression of Fas ligand leading to increased monocyte apoptosis, but this does not occur in activated macrophages expressing ERα [134]. Androgen receptor expression on/in human monocytes is higher in men than in women [153–155]. While ERα and ERβ bind estrogen with similar affinity, the use of selective ER agonists revealed that while ERα stimulates T cell proliferation, ERβ promotes apoptosis [156, 157].

Estrogen also activates B cells in response to infection or damaged-self (i.e., autoimmunity) to increase antibody/autoantibody levels, thereby contributing to IC formation. In contrast, androgens reduce B cell synthesis of antibody/autoantibody in men [62, 135–138, 158]. Although increased antibody levels protect women from infections, they increase the risk for autoimmune diseases. In cell culture studies and animal models, estrogen has been found to activate innate immune cells like dendritic cells, stimulate T cell proliferation, skew the immune response toward Th2 and M2, and increase IL-4 and TGF-β levels [40, 138, 159–162], all of which are able to increase remodeling and fibrosis in lung cells. Far less, research has been conducted on the effect of androgens [118, 163, 164].

Menopause is defined as 1 year without a menstrual period [165–167] and occurs in Western cultures between age 49 and 55 [165, 166]. Decreases in estrogen (i.e., estradiol) occur in the last 6 months before menopause and thereafter [167]. In contrast, testosterone gradually decreases with age in both sexes [168]. Understanding changes in immune function following menopause are complicated by changes that occur because of aging [169, 170]. Menopause is associated with a decrease in T cell activity and an increase in IL-1, IL-6, and TNF levels [171–173]. That low estrogen increases proinflammatory cytokines is consistent with the known ability of high doses of estrogen via ERα to downregulate NFκB, Th1 responses, IL-1, and IL-6 in various human and murine cells [148, 150, 174–176]. Thus, after menopause, the protective role of estrogen is lost allowing increased proinflammatory cytokine levels while at the same time, antibody/autoantibody levels continue to rise (Fig. 1). This may promote certain autoimmune diseases like rheumatoid arthritis and Sjögren’s syndrome that peak after 50 years of age [177]. In contrast, a gradual decline in androgen levels past age 50 decreases inflammation in men without the elevated antibodies/autoantibodies found in women.

Women have higher ER expression in their arteries than men, which decreases after menopause [178]. ERβ signaling increases arterial tone and blood pressure, while ERα reduces vascular injury [179, 180]. Animal models of heart disease demonstrate that testosterone is responsible for adverse cardiac remodeling in the myocardium of males that leads to heart failure [6, 118, 181] and that estrogen signaling via ERα, in particular, prevents cardiac myocyte hypertrophy and fibrosis in females by inhibiting remodeling cytokines and collagen synthesis [180]. These findings suggest that ERβ may promote the negative effects of estrogen in pulmonary arteries in PAH, while ERα may be protective.

## Sex differences in the immunopathology of PAH

PAH can occur at any age but typically between age 36 and 47 [9, 17, 18, 21, 95], suggesting that estrogen drives the pathogenesis of disease because PAH is a chronic condition that takes years to develop. SSc is also typically diagnosed prior to menopause in women, while men with SSc are diagnosed at an older age than women [34]. Recall that the sex ratio of women to men with SSc prior to menopause is estimated at 15:1 while it lowers to 2.4:1 after menopause [34–36], suggesting that estrogen promotes SSc. Surprisingly, circulating estradiol was found to reduce PAH in female Sprague Dawley rats that had undergone ovariectomy and estrogen replacement [182]. However, the study did not distinguish the role of ERα vs. ERβ in mediating the effect of estrogen. Estrogen via ERβ signaling has been shown to regulate arterial tone and blood pressure, while ERα protects against vascular injury, remodeling and fibrosis, and atherosclerosis [5, 6, 39, 40]. It is possible that current animal models of PAH do not fully recapitulate the clinical picture [183–185].

Male rats with hypoxia-induced pulmonary hypertension had more severe disease than females [186]. This finding is similar to humans, where men with PAH-associated SSc/dcSSc have more severe disease and worse survival [19, 20, 35, 37, 38, 56]. In this model, pulmonary vascular remodeling was reduced in males by 17β-estradiol and required ERα and ERβ signaling [187]. 2-Methoxyestradiol, a major non-estrogenic metabolite of estradiol, was also found to protect male rats from monocrotaline-induced PAH by decreasing pulmonary arterial hypertrophy and reducing proliferative and inflammatory responses in the lung [188]. More research is needed in animal models of PAH in order to determine the role of sex hormones in the immunopathogenesis of disease.

Importantly, recent research provides evidence that ERα via the estrogen response element is able to bind the BMPR2 promoter and modulate its activity [189]. In animal models of PAH, BMPR2 expression on lymphocytes and the pulmonary system was significantly lower in females than in males [189], suggesting that ERα may promote PAH by downregulating BMPR2. Additionally, CYP1B1, an estrogen-metabolizing enzyme, was expressed 10× less in females with BMPR2 mutations than those without [125, 190]. These data suggest that BMPR2 protects women from PAH and estrogen/ERα decreases its expression. Women with mutations/polymorphisms in BMPR2 may have a greater risk of developing PAH. Additionally, the central role for estrogen in driving a Th2 response that activates B cells and elevates autoantibody and IC levels could increase the prevalence of CTD/autoimmune disease-associated PAH in women (Fig. 1) [9, 26].

A study of 63 SSc patients that presented with isolated pulmonary hypertension found that menopause increased the risk of developing PAH, particularly in the subgroup of women with CREST (these patients also have Raynaud’s syndrome) [191]. CREST syndrome is associated with the development of SSc-associated PAH at a later age (i.e., menopause). Hormone replacement therapy improved lcSSc-associated PAH in post-menopausal women [192]. All 20 lcSSc patients that received hormone replacement did not develop PAH whereas 8/41 (19.5%) lcSSc patients that did not receive therapy developed PAH after menopause [192]. These data suggest that menopause worsens disease in women with PAH. Raynaud’s syndrome, which is associated with SSc and SSc-associated PAH, occurs more often in women than in men [55]. ANA present in Raynaud’s are believed to contribute to the pathogenesis of disease by contributing to IC deposition in vessels [58, 62]. Estrogen is thought to play a role in the pathogenesis of Raynaud’s syndrome, with the incidence of vasospastic reactions increasing during the pre-ovulatory period and with estrogen administration [193]. However, contradictory findings on the effect of sex hormones and hormone replacement therapy exist for Raynaud’s syndrome. Part of the difficulty in interpreting the effect of hormone replacement therapy on autoimmune or other chronic inflammatory diseases like PAH is that different combinations of hormones are used in therapies and they are administered to women at different ages.

There are a number of reasons why clinical and animal model data may appear to be contradictory for PAH, SSc, and Raynaud’s syndrome. High doses of estrogen have opposite effects on inflammation compared to low doses [5, 23], and so hormone replacement therapy may not exactly mimic pre-menopause conditions. Estrogen decreases inflammation by increasing regulatory Th2, Treg, IL-10, and TGF-β [62]. A recent report found that depletion of Treg in animal models of pulmonary hypertension increased disease in female rats [194], indicating the importance of Treg in preventing vascular injury in females. However, high doses of estrogen increase inflammation, as occurs during pregnancy and with hormone replacement therapy. These opposing effects of estrogen are likely due to differential signaling through ERα, ERβ, GPR30, and/or other steroid receptors. Estrogen, but not testosterone, increases antibody/autoantibody levels and thereby promotes ICs. This is true even for low doses of estrogen that occur after menopause. IC deposition activates complement, innate immune mechanisms like TLR4, decreases Treg, and increases inflammation [5]. Thus, one possible explanation for contradictory data is that estrogen in females is increasing inflammation via autoantibody/IC deposition. So even though estrogen levels drop significantly after menopause, low levels of estrogen continue to drive autoantibody diversity. Thus, estrogen may increase the incidence of PAH and SSc-associated PAH in women by increasing antibody/autoantibody production, even following menopause, leading to IC deposition, tissue damage, and inflammation (Fig. 1). In contrast, testosterone increases cell-mediated inflammation via mast cells and macrophages that release proinflammatory and profibrotic cytokines (i.e., IL-1β) that could be induced or amplified by infections (Fig. 1).

## Knowledge gaps

Understanding how sex differences in the immune response to infection and autoimmunity influence PAH subgroups may provide important information about the pathogenesis of disease (Fig. 1). Future studies should analyze data on individual PAH subgroups according to sex. Heritable cases should be analyzed separately because the pathogenesis of disease differs (i.e., genetic vs. environmental). Additionally, combining PAH groups according to sex may increase the statistical power of studies vs. analyzing data only by PAH subgroup [23, 75]. Future studies also need to consider age in the analysis of PAH. That the sex ratio of women to men with SSc is 15:1 prior to 50 years of age but lowers to 2.4:1 after 50 [35, 36] indicates the importance of analyzing data by sex and age using age 50 as a cutoff. Age 50–55 is most commonly used to study the effect of menopause when the actual menopause status of the subject is not known. Very little data exists for the effect of andropause on disease in men. Aging affects both the immune system and host tissues due to a decline in cell function with age and changing levels of sex hormones. Although substantial therapeutic advances have occurred over the past 25 years for PAH patients [195], therapies could be improved by considering sex and age differences.

## Conclusions

Most PAH registries report an overall female predominance of 2–4 over men. However, CTD-associated PAH is as high as 9:1 female to male while some other PAH subtypes occur more often in men. More research examining sex differences in PAH is needed. Analysis of all PAH subtypes by sex and age, with specific efforts to identify mechanistic differences, may provide potential treatment targets.

## Abbreviations

AIHA: Autoimmune hemolytic anemia
ANA: Anti-nuclear autoantibody
BMPR: Bone morphogenic protein receptor
CAD: Coronary artery disease
CTD: Connective tissue disease
CVD: Cardiovascular disease
dcSSc: Diffuse cutaneous SSc
EC: Endothelial cell
ER: Estrogen receptor
FGF: Fibroblast growth factor
IC: Immune complex
IGF-1: Insulin-like growth factor-1
IL: Interleukin
lcSSc: Limited cutaneous SSc
NIH: National Institutes of Health
PAH: Pulmonary arterial hypertension
PDGF: Platelet-derived growth factor
SSc: Systemic sclerosis
STAT: Signal transducer and activator of transcription
TGF: Transforming growth factor
Th: T helper-type cells
TIMP: Tissue inhibitors of MMP
TNF: Tumor necrosis factor
Tregs: Regulatory T cells
VEGF: Vascular endothelial growth factor
VSMC: Vascular smooth muscle cell
